# Recurrent Giant Myofibroblastic Sarcoma of Neck in a Teenager: Overcoming Treatment Challenges

**DOI:** 10.1007/s12070-023-03752-y

**Published:** 2023-05-06

**Authors:** Diksha Dinker, Keshava Rajan, Naveena A. N. Kumar, Vishwapriyan Godhkini

**Affiliations:** 1 Department of Surgical Oncology, Manipal Comprehensive Cancer Care Centre, Kasturba Medical College, Manipal Academy of Higher Education(MAHE), Manipal, Karnataka 576401 India; 2 Department of General Pathology, Kasturba Medical College, Manipal Academy of Higher Education, Manipal, Karnataka 576104 India

**Keywords:** Head and neck sarcoma, LGMS, Trimodality treatment, Recurrent sarcoma

## Abstract

Sarcomas are rare tumors arising from a variety of mesenchymal tissues which are even rarer in head and neck region amounting 1% only of the 5% of sarcomas. About 80% of head and neck sarcomas originate in soft tissue while the remaining 20% arise from bone (Cormier and Pollock in J Clin 54:94−109, 2004). One among the commonly presenting variants of sarcomas in head and neck that our patient was diagnosed with is Low grade myofibroblastic sarcoma (LGMS). These even though uncommon have a predilection for head and neck region particularly the tongue (Mentzel et al. in Am J Surg Pathol 22:1228−38, 1998; Cai et al. in Virchows Arch 463:827−36, 2013; Meng et al. in Chin Med J 120:363–9, 2007; Demarosi et al. in Oral Surg Oral Med Oral Pathol Oral Radiol Endod. 108:248–54, 2009). LGMS was reclassified as a distinct entity by the WHO classification of soft tissue tumors in 2002 (Qiu et al. in Oncol Lett 9:619−25, 2015). Oncological resection is the mainstay of treatment. In case of bulky tumor, resection and reconstruction would be challenging. We report a recurrent LGMS of 15-year-old girl who presented with a ginormous soft tissue swelling in her left neck region which made her day-to-day activities strenuous and made her non ambulatory and the line of treatment executed in order to get her back on feet, healthy & free of the debilitating tumor.

## Case Presentation

A young adolescent girl presented to us with complaints of gradually increasing painful swelling with ulcer and bleeding from tumor surface in her left neck region which made her daily activities close to impossible with the amount of strain it caused along with restricting her movement and making her non ambulatory in spite of taking both surgical and medical line of treatment for the same over a course of 3 years.

The first presentation of the swelling was reported way before the patient presented to us. First history of swelling in the same region was three years ago, for which she underwent excision at other hospital. Patient quickly encountered symptoms of local recurrence in the following months and underwent another surgery in 2018, wherein excision of the swelling was done & remained symptom free post that for a brief period only as she displayed symptoms of local recurrence yet again in the year 2020.The previous excision biopsy reported to be low grade myofibroblastic sarcoma. In view of small size and recurrent nature, she was started on metronomic chemotherapy and planned for reassessment. However, in view of COVID-19 pandemic, she defaulted and presented with bulky tumor. She has received hemostatic radiotherapy and palliative chemotherapy just prior to our consultation.

**Fig. 1:**
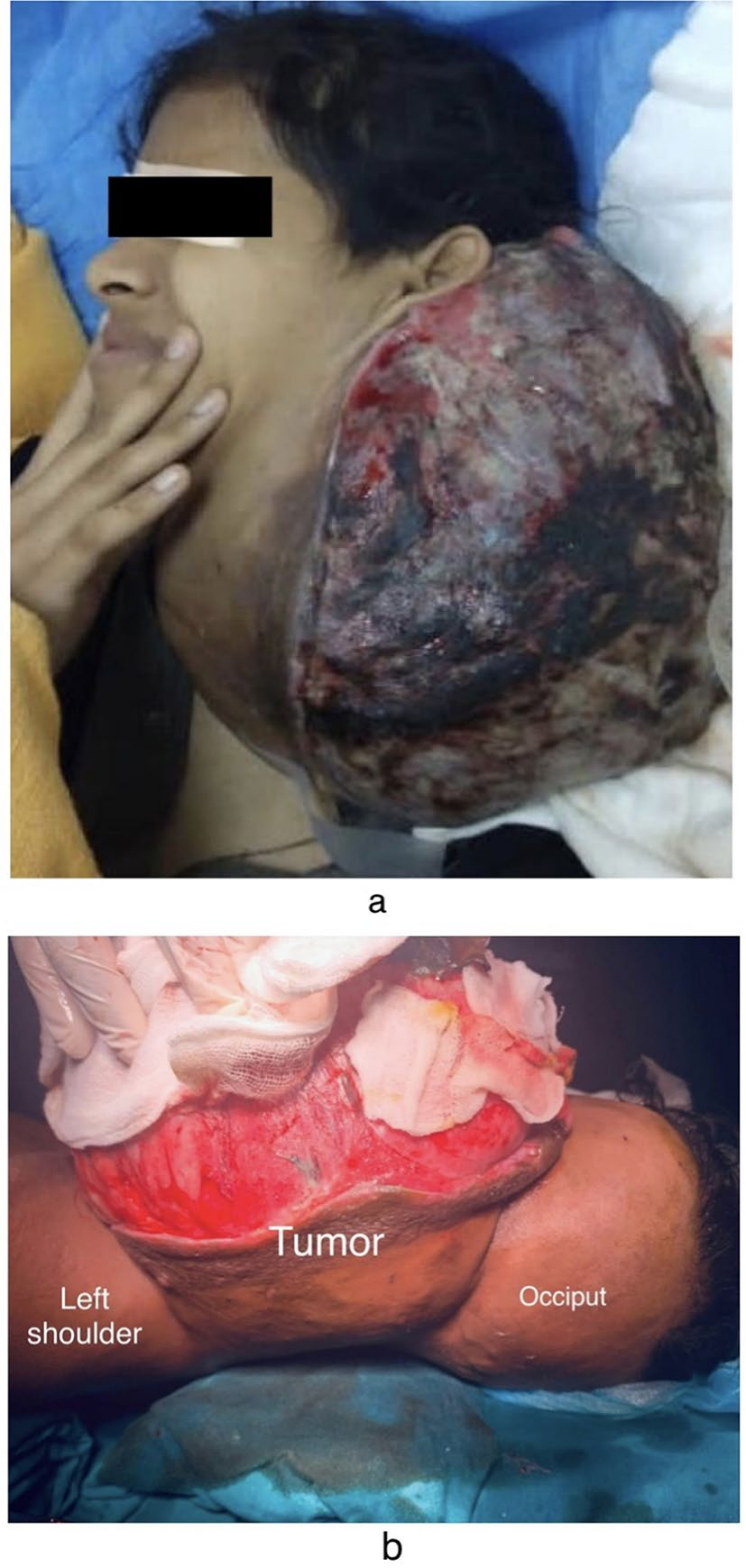
**a**, **b** Clinical presentation of the tumor

She was bedridden in view of bulky tumor. On examination of the left cervical region, a 23 × 25 cm well defined fixed swelling with bleeding ulcer was noted with erythema in the surrounding skin (Fig. 1a, b). The tumor was occupying more than 50% of neck circumference and extending vertically from mastoid region to supraclavicular region. The size of the swelling caused the external ear to be pushed anteriorly. There were no palpable cervical lymph nodes. No signs of facial nerve palsy were displayed.

**Fig. 2:**
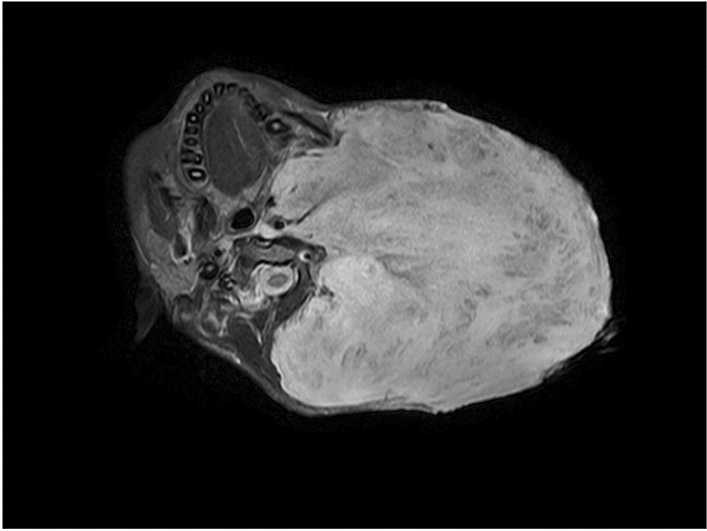
MRI showing the extensions of the lesion in axial plane

On investigation, MRI of neck (Fig. 2) showed 25 × 20 cm lesion infiltrating left sternocleidomastoid muscle extending superiorly up to parotid, external auditory canal and mastoid area. The facial nerve could not be localized distinctly from the tumor. The tumor was extending into parapharyngeal and pharyngeal space, displacing larynx and hypopharynx, pushing left carotid artery medially and there was involvement of left internal jugular vein (IJV) with collateralization of flow with prominent anterior jugular venous arch. The lesion was also seen to infiltrate left peri vertebral & posterior cervical space along with its encroachment into the overlying skin. Contrast enhanced computed tomography did not show any distant metastasis.

## Treatment

**Fig. 3:**
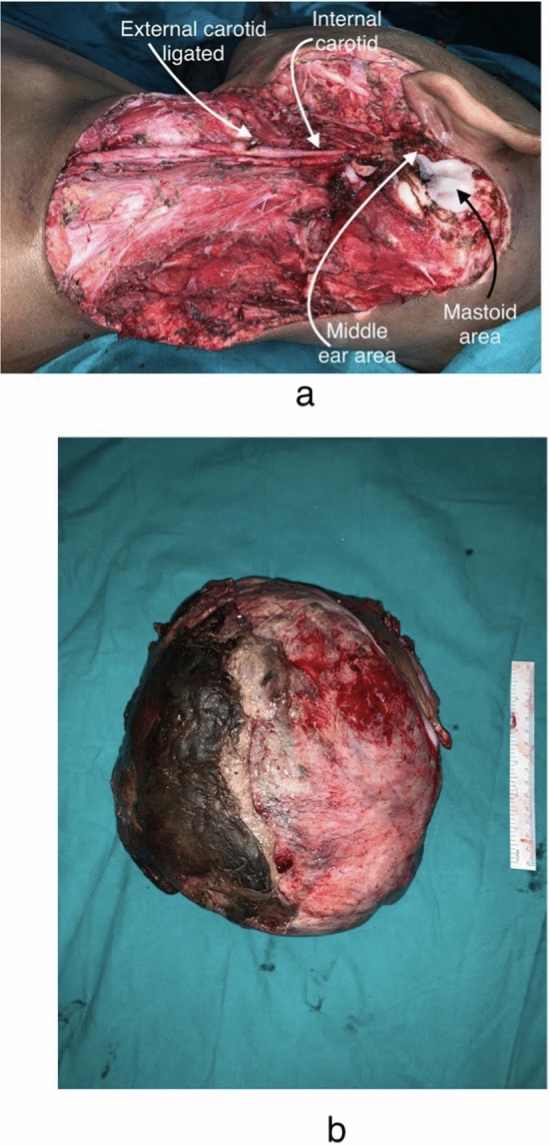
**a** Tumor bed, **b** Resected specimen

**Fig. 4:**
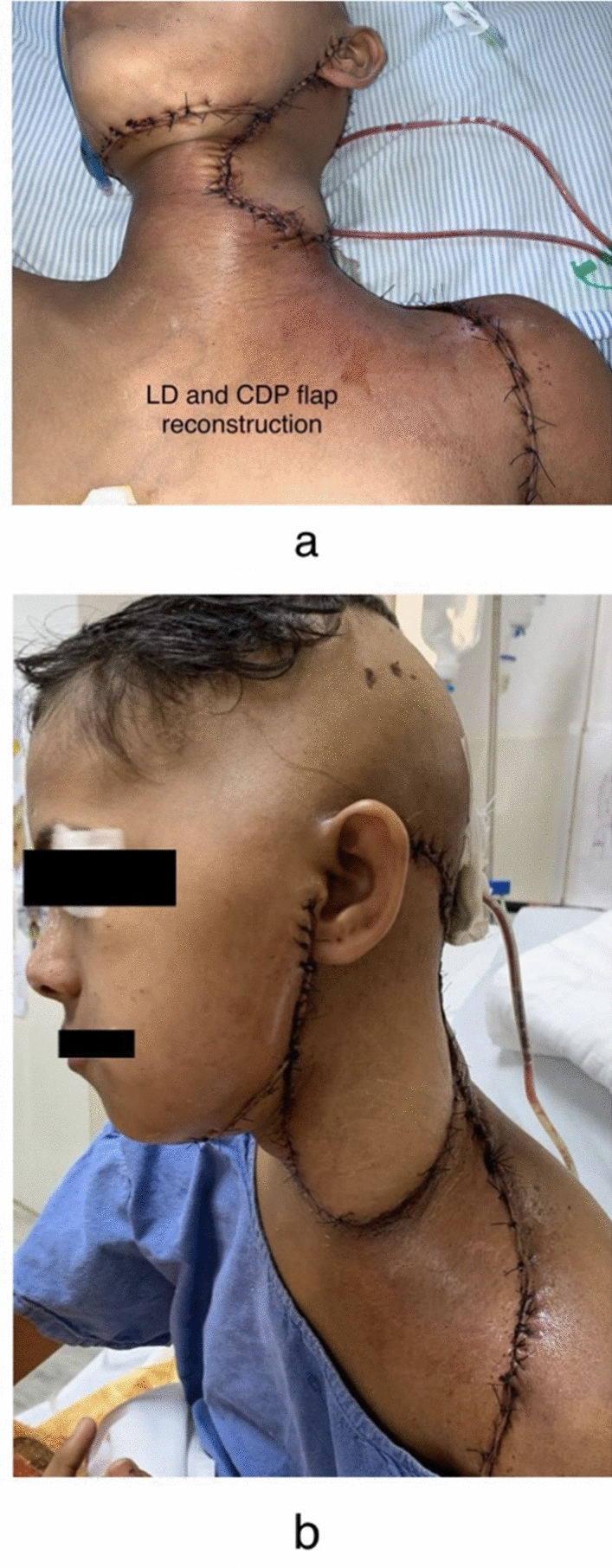
**a**, **b** Latissimus Dorsi myocutaneous flap and cervico-deltopectoral flap

Following meticulous planning, the patient was planned for semi-emergency surgery in view of tumor bleeding following blood transfusion and optimization. The possibilities of R2 resection near external auditory canal, facial nerve paresis/palsy and ligation of left IJV was kept in mind and explained to parents. The first step of surgery was to dissect the carotid sheath. Left common carotid artery was dissected and freed. The external carotid artery was ligated. The dissection continued towards skull base by safeguarding internal carotid artery. Left IJV was ligated superiorly and inferiorly after getting control near skull base and supraclavicular fossa respectively. The posterior dissection started with adequate tumor free margin. The last part of surgery was at external auditory canal and mastoid area. We could not identify Facial nerve trunk separately from the tumor in view of large tumor size and previous surgery scar, which made dissection was difficult. Gross total resection was done and remaining soft tissue at middle ear area was completely removed by piece meal technique. (Fig. 3a, b). The Latissimus Dorsi myocutaneous flap of 20 × 12 cm size was harvested for primary defect. The remaining defect was covered by ipsilateral and contralateral cervico-deltopectoral flap (Fig. 4a, b).

On postoperative day 3, she had sudden onset of massive bleeding at drain. She was taken to emergency operation room. On exploration, there was bleeding from lateral wall of carotid artery, which was repaired using 5–0 prolene sutures. Again after 2 days, she had one more episode of massive bleeding and was taken to emergency OR. This time, left common carotid artery was ligated. Postoperatively, Left tarsorrhaphy was done and facial muscle physiotherapy was started in view of facial nerve palsy. There were no neurological symptoms related to left carotid ligation and was discharged after 1 week.

## Outcome & Follow-up

**Fig. 5:**
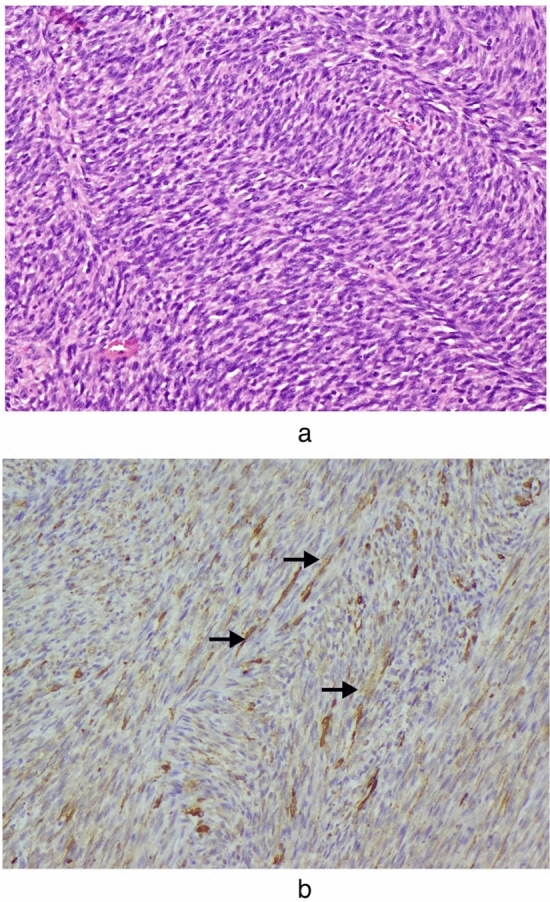
**a** H&E 100X: Shows diffuse & focal storiform proliferation of spindled tumor cells with mild to moderate nuclear atypia. **b** SMA 400x: The myofibroblastic cells show a "tram-tracking" pattern of staining[arrow] with Smooth Muscle Actin

The final histopathology reported to be Low grade Myofibroblastic Sarcoma which showed spindle shaped cells in a sheet like and fascicular pattern with indistinct borders (Fig. 5a, b).

The patient thereafter received adjuvant radiotherapy and remains disease free at 24 months allowing her to carry out her daily activities which were compromised earlier due to the disease.

## Discussion

LGMS is a malignant tumor of myofibroblastic origin occurring at submucosal and subcutaneous locations. These generally affect middle aged patients with an average age of 40 years but can affect any age group and has a male predominance [7]. LMGS has been described at several anatomical locations of the human body, the head and neck region is frequently affected with rare ophthalmic involvement. It’s locally aggressive, may recur, sometimes metastasize or even progress to a higher grade. LGMS can be focally contained, still the majority infiltrate into the adjacent fibrous tissue, fat, muscle or even bone. Recurrent tumors tend to exhibit increased pleomorphism or display areas of higher-grade malignancy.

A study by Yamada et al. [4], reported a 38.2% recurrence rate among 38 cases of LGMS, which is one of the highest values cited from nasal cavity/paranasal sinus LGMSs, the second highest after cases of the jawbone, followed by the deep tissue space. The recurrence rate for tumors of < 3 cm in size is 21.4%, but for tumors > 3 cm, the recurrence rate increases to 46.2%. In the aforementioned study, the common sites affected by LGMS, after the tongue, are the maxilla and palate, the mandible, the nasal/paranasal cavity and the deep tissue spaces, including the parapharyngeal space [8].

Even though there’s no validated treatment protocol for this tumor and the local aggressive presentation, bleeding and the history of multiple local recurrences of this tumor in our patient may have been intimidating for the surgeon’s knife at first, we went ahead with the wide local excision of the tumor followed by adjuvant radiotherapy.

Owing to the rarity of LGMS, the optimal treatment is still unclear. Like other soft-tissue sarcomas, LGMS is primarily treated with surgical excision [9]. For patients without clear tumor margins, radiotherapy is an option. Hence, surgical resection is the primary treatment modality for LGMS. Chemotherapy and radiotherapy showed limited effects on patient survival. Adjuvant application of chemotherapy and/or radiotherapy in patients with LGMS was not correlated with improved survival. Therefore, chemotherapy and/or radiotherapy should not be routinely performed to treat LGMS patients with negative margins. For patients with positive margins or recurrent disease, chemotherapy and/or radiation could be an alternative treatment but cannot replace the importance of surgical intervention for the same [10].

The combination of surgical excision along with adjuvant radiotherapy instead of treating it only with palliative chemotherapy and radiotherapy improves not only the quality of life, but also ensures a better disease-free survival rate and keeps the recurrence at bay.

To conclude, combination of wide excision and plastic reconstruction followed by adjuvant radiotherapy provide best possible disease-free survival and better quality of life in a patient with recurrent, giant Myofibroblastic Sarcoma of Neck.
